# High Efficiency Focusing and Vortex Generator Based on Polarization-Insensitive Gallium Nitride Metasurface

**DOI:** 10.3390/nano11102638

**Published:** 2021-10-07

**Authors:** Zhitong Sun, Bijun Xu, Bairui Wu, Xiaogang Wang, Hao Ying

**Affiliations:** 1 School of Sciences, Zhejiang University of Science and Technology, Hangzhou 310023, China; a1441901048@163.com (Z.S.); 211909702006@zust.edu.cn (B.W.); wxg1201@163.com (X.W.); 2 Department of Physics, Hangzhou Dianzi University, Hangzhou 310012, China; yinghao@hdu.edu.cn; 3 Department of Physics, Zhejiang University, Hangzhou 310023, China

**Keywords:** highly efficient, polarization-insensitive, GaN metasurface

## Abstract

In this paper, two polarization-insensitive Gallium Nitride (GaN) metasurfaces based on a dynamic phase for adjusting the wavefront are proposed. Specifically, we obtained the target phase to satisfy some design conditions by changing the structural parameters at the nanoscales. Under the irradiation of linearly polarized (LP) light and circularly polarized (CP) light, respectively, one of the metasurfaces can generate a focused beam with an efficiency of 84.7%, and the other can generate a vortex beam with a maximum efficiency of 76.6%. Our designed metasurfaces will have important applications in optical communication, holographic projection, and particle capture.

## 1. Introduction

Traditional lenses made of natural materials are the main means of beam focusing. However, the inflexibility of natural materials may lead to inefficient and oversize lenses [1,2,3,4]. At the same time, traditional lenses are not suitable for some flat optical devices because of their convex structure. Therefore, it is particularly important to design lenses with a small volume and high efficiency [5,6,7,8]. In comparison to natural materials, metamaterial, as a kind of artificially designed composite material, has supernormal physical properties such as negative refraction and electromagnetic stealth, which natural materials do not have [9,10,11]. In recent years, metasurface, a 2D metamaterial, has attracted more and more attention because of its subwavelength size, planar shape, and multi-function integration [12,13,14,15,16]. Since each nanoscale of a metasurface can independently control the wavefront of the incident beam, the designed purpose can be achieved through different arrangements of the nanoscales, and the incident beam can be reshaped with a large degree of freedom of motion [17,18,19,20,21].

In this paper, a method has been elaborated for designing two types of GaN metasurfaces based on the dynamic phase. One of the metasurfaces can generate a focused beam, while the other can generate a vortex beam. Compared with the focused beam, vortex beams carry orbital angular momentum (OAM) because of the spiral phase factor exp(imθ) [22,23]. The vortex generator acts as a combination of a focusing lens and a spiral phase plate. It is expected to play an important role in holographic projection [24,25], optical communication [26,27] and optical acquisition [28,29].

Compared with Si and TiO_2_ metasurfaces [30,31], our metasurfaces use the third-generation semiconductor material GaN, which can perform beam regulation more efficiently. At 630 nm wavelength, the simulation shows that a focused beam’s efficiency is 84.7%, while the vortex beam’s maximum efficiency is 76.6%. The simulation results proved the superiority of GaN and are also consistent with the theoretical analyses. Due to the advantages of high power and high bandwidth, the GaN metasurface can be widely applied in industrial communication fields, and it will lead the third-generation semiconductor market [32,33].

## 2. Structure Design

**Figure 1:**
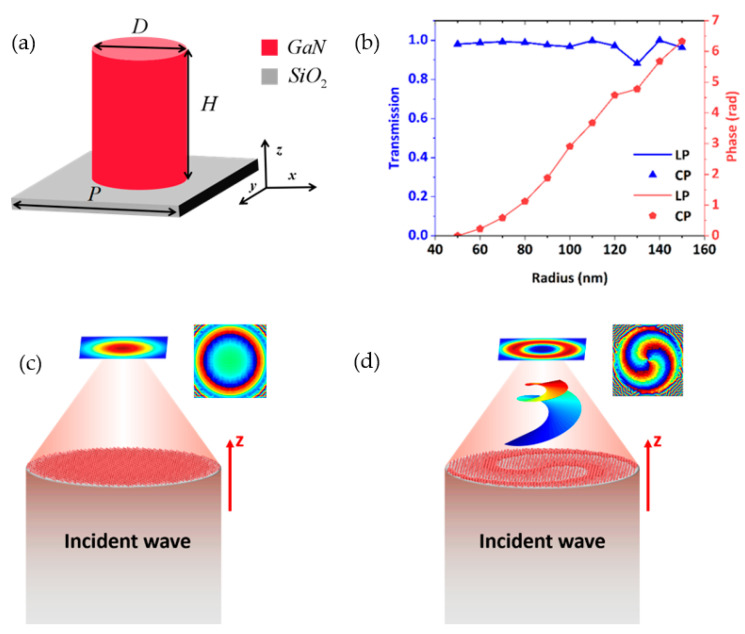
(**a**) Schematic of the nanoscales. (**b**) Transmission and phase of the nanoscales. (**c**) Schematic diagram of the metasurface with focused beams, and phase distribution of the near field. (**d**) Schematic diagram of a vortex generator, and phase distribution of the near field.

The dynamic phase is mainly related to the refractive index of the material and the geometry of the nanoscale of the metasurface. Figure 1a schematically depicts a dielectric metasurface unit consisting of a GaN nanoscale embedded on a SiO_2_ substrate. To further obtain the phase distribution, the numerical simulation of the nanoscale is performed using the commercially available three-dimensional finite difference time domain (FDTD) solver from Lumerical. The FDTD method uses polarization along the *x*- and *y*-axis to obtain the transmission coefficients of a series of nanoscales. The wavelength of the incident light is *λ* = 630 nm, and the period is *P* = 400 nm. In addition, the diameter *D* of the dielectric column is in the range of 100 to 300 nm, and the height is fixed at *H* = 700 nm. Perfectly matched layers (PMLs) were used in the *z*−direction, and periodic boundary conditions (PBCs) were applied in the *x* and *y* directions. Figure 1b shows the functional relationship between the transmission T and the phase, with the radius R of the nanoscales. It can be seen that the transmissions are high. All the transmissions are greater than 87% and most of them are greater than 95%. As shown in Figure 1b, the phase spans from 0 to 2π independently, so an arbitrary phase can be obtained by selecting the appropriate R. The plane wave propagates along the +*z* direction, and by adjusting R, the transmitted wave refracts along the specified direction to produce a focused beam or vortex beam. The phase and transmission curve distributions are almost identical when LP and CP are incident, respectively (Figure 1b). This provides a prerequisite for us to design polarization−insensitive structures. Figure 1c,d are schematics that metasurfaces can generate a focused beam and a vortex beam, respectively. They show that the metasurfaces have a strong capacity to control the incident wave.

## 3. Theory and Results Analysis

**Figure 2:**
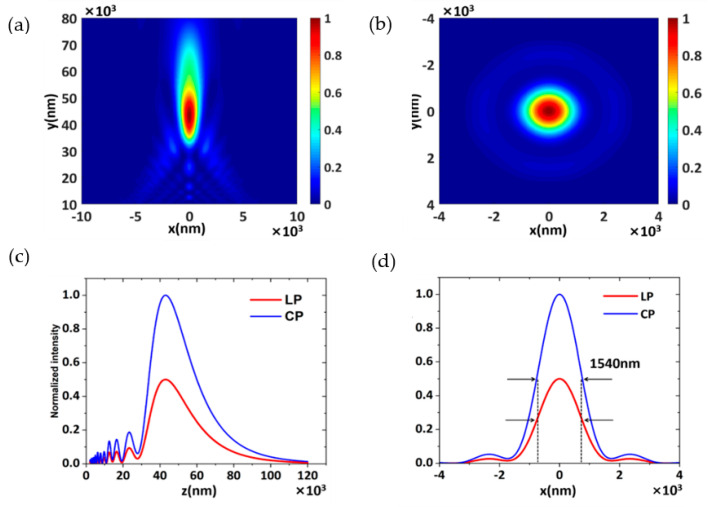
(**a**) The far−field normalized intensity distribution. (**b**) The profile of the focal point. (**c**) The far−field normalized intensity distribution curve. (**d**) The normalized intensity of the focal point.

Figure 1c presents a schematic diagram of the metasurface with the focused beam. It is made up of hundreds of GaN nanoscales. The phase of GaN nanoscales in the transverse plane (*x*, *y*) is expressed by:

(1) $$\varphi =2\pi (f-\sqrt{x^{2}+y^{2}+f^{2}})/\lambda$$

where *φ* is the focusing phase distribution, *λ* is the wavelength of the incident beam, and *f* is the focal length. In this study, the focal length was set to *f* = 50,000 nm, *λ* = 630 nm. To better demonstrate the focus characteristics of the metasurface, we calculated the far-field normalized intensity distribution (Figure 2a) and the normalized intensity of the focused beam on the metasurface in the *x*–*y* plane, and at the focus, *z* = 50,000 nm (Figure 2b). Figure 2c reveals the normalized intensity distribution curve of focusing in the +*z* direction when the incident light is LP and CP, respectively. The results show that there is a difference of two times between the values of the two focusing intensity curves, because CP can be equivalent to the combination of two orthogonal LP with a phase difference of π/2. In this case, each orthogonal source has an amplitude of 1, this means that Ex and Ey are both 1. This implies that $\left|E\right|=\sqrt{2}(V/m)$. A power transmission located in front of the sources would return 1 (not 2) because the transmission function is normalized to the sum of the source power from all sources. Thus, the strength of CP is twice the strength of the LP, resulting in this consequence (Figure 2c). As shown in Figure 2c, although the intensity performance is inconsistent, the focal point position and the intensity distribution of the focused beam are identical, which verifies the polarization insensitivity of the structure. Figure 2d shows the normalized intensity curve distribution at the focal point. It can be seen that the full width of the half-maximum intensity of both curves is *ω* = 1540 nm, and the diffraction-limited full width at half maximum (FWHM=λf/L) is 1540 nm. Therefore, our results are obtained close to the diffraction limit. The focusing efficiency of the metasurface is 84.7%, which is calculated by dividing the intensity of the light at the focal point by the intensity of the incident beam. The above results show that the metasurface has great optical control ability.

As shown in Table 1, we compared the focusing efficiency of various metasurfaces in different studies, which implies that our structure has great advantages regarding focusing efficiency. In addition, GaN has the characteristics of low cost and high efficiency compared with other materials.

**Figure 3:**
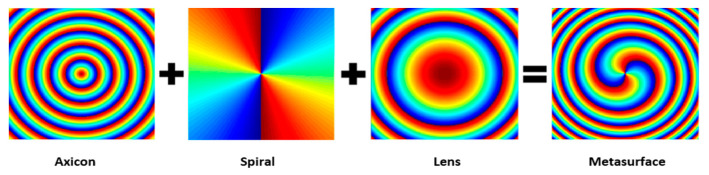
The formation of the metasurface phase profile by superposing the phase profiles of an axicon, spiral phase plate, and Fourier transformation lens.

In addition, we used designed units to construct metasurfaces that generated vortex beams. Theoretically, an ideal vortex beam can be obtained by the Fourier transform of the ideal Bessel beam, which is as follows [38]:

(2) $$E_{b}(r,z)=J_{l}(k_{r}\rho )\cdot \exp(im\theta +ik_{z}z)$$

where $\sqrt{k_{r}{}^{2}+k_{z}{}^{2}}=k=2\pi /\lambda$ is the wave vector at the incident wavelength, $J_{l}$ is the first class of *l*—order Bessel functions, *r* is the polar coordinate in the beam cross-section, $k_{r}$ is the radial wave vector, and $k_{z}$ is the longitudinal wave vector. The ideal vortex beam is generated by the Fourier transform of the Bessel–Gaussian (BG) beam [39], and then:

(3) $$E_{b}{}_{g}(r,z)=J_{l}(k_{r}\rho )\cdot \exp(im\theta -\rho^{2}/\omega_{0}{}^{2}+ik_{z}z)$$

where $\omega_{0}$ is the width of the light field. The ideal vortex beam can be obtained by the following steps. Firstly, the Gaussian beam is converted into a Laguerre Gaussian (LG) beam by a spiral phase plate. Secondly, an axicon is utilized to convert the LG beam into a BG beam. Thirdly, using a Fourier lens can transform the BG beam into an ideal vortex beam. Subsequently, the phase profile of the vortex generator can be expressed as [40]:

(4) $$\varphi (x,y)=\varphi_{a}(x,y)+\varphi_{b}(x,y)+\varphi_{c}(x,y)$$

where:

(5) $$\varphi_{a}=-2\pi \frac{\sqrt{x^{2}+y^{2}}}{p}$$

(6) $$\varphi_{b}=l\cdot \arctan(x/y)$$

(7) $$\varphi_{c}=\frac{-\pi (x^{2}+y^{2})}{\lambda f}$$

where (*x*, *y*) is the coordinate of each nanoscale, *P* is the periodic constant of the axicon, and *l* is the topological charge. Figure 3 shows an example of the phase superposition of Equations (5)–(7) at *l* = 2, *P* = 4000 nm.

**Figure 4:**
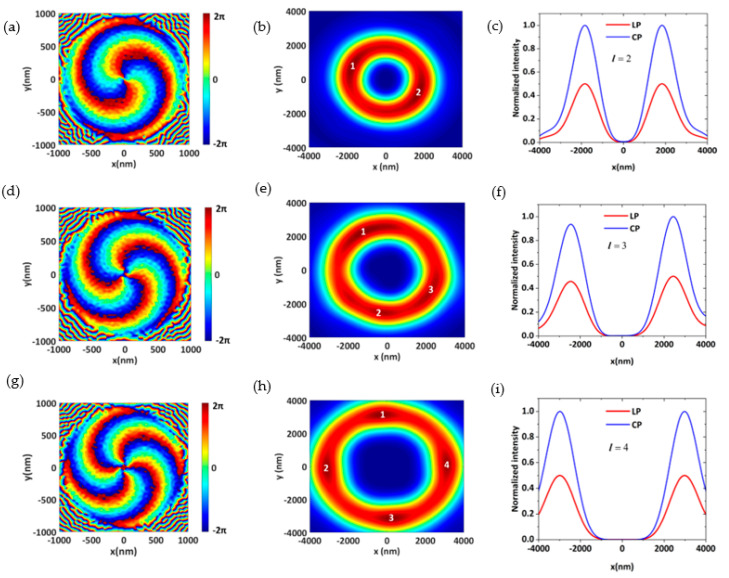
(**a**,**d**,**g**) Phase distribution of the near−field. (**b**,**e**,**h**) The profile of the focal point. (**c**,**f**,**i**) The normalized intensity of the focal point.

Figure 1d is a schematic diagram of the vortex generator and phase distribution of the near field (*l* = 2). According to Equation (6), we constructed the metasurface of topological charges *l* = 2, *l* = 3, and *l* = 4. Figure 4a,d,g show the near−field phase distribution of the vortex generator. As shown in Figure 4b,e,h, the phase singularity of the vortex center generates the distribution of the center hole, and the topological charge number of the vortex beam can be distinguished, which verifies the good performance of the device. The efficiency of vortex beam generation is 76.6%, 71.4%, and 63.9%, respectively. When the incident light is LP and CP, the vortex generator can produce almost the same vortex beam (Figure 4c,f,i). However, due to the different intensity of the incident beam, the intensity of vortex beams will be approximately two times higher. This shows that the structure has good polarization insensitivity.

## 4. Conclusions

In this paper, we proposed two metasurfaces based on the dynamic phase to generate a focused beam and a vortex beam at the incident wavelength of 630 nm. The advantage of the dynamic phase is that it requires less polarization of the incident light. The focus intensity curves of the beams are highly coincident when LP and CP are incident, respectively, which verifies the polarization insensitivity of the structure. Therefore, when different polarization light beams are incident, we do not need to redesign the metasurfaces or change the polarization of the light. Meanwhile, the designed metasurfaces have the advantage of high efficiency, which can be applied into the field of communication. However, there is a certain deviation between the theoretical focal point and the simulated focus, which is caused by the small size of the structure and the small number of nanoscales used in simulations. Note that the problem can be solved by changing the size of the metasurface. Be aware that the structure itself has low wastage due to electric or magnetic dipole resonances. To sum up, the metasurfaces we designed have strong capabilities for beam regulation, and can provide an efficient approach for optical control, which has potential in many fields, ranging from communication to artificial intelligence.

## Figures and Tables

**Table 1:** Summary of our result and other references.

References	Efficiency	Material	Wavelength
Our result	85%	GaN	630 nm
[4]	23%, 39%, 54%	Au	532 nm, 632.8 nm, 780 nm
[34]	27%	Au, MgF_2_	900 nm
[35]	20%	ITO	850 nm
[36]	60%	TiO_2_	532 nm
[37]	20%	Copper	30 mm

## Data Availability

Data are contained within the article.
